# *Robinia pseudoacacia* L. (Black Locust) Leaflets as Biomonitors of Airborne Microplastics

**DOI:** 10.3390/biology12121456

**Published:** 2023-11-22

**Authors:** Mehriban Jafarova, Lisa Grifoni, Monia Renzi, Tecla Bentivoglio, Serena Anselmi, Aldo Winkler, Luigi Antonello Di Lella, Lilla Spagnuolo, Julian Aherne, Stefano Loppi

**Affiliations:** 1 Department of Life Sciences, University of Siena, 53100 Siena, Italy; mehriban.jafarova@student.unisi.it (M.J.); l.grifoni2@student.unisi.it (L.G.); luigi.dilella@unisi.it (L.A.D.L.); stefano.loppi@unisi.it (S.L.); 2 Istituto Nazionale di Geofisica e Vulcanologia, 00143 Rome, Italy; aldo.winkler@ingv.it (A.W.); lilla.spagnuolo@ingv.it (L.S.); 3 Department of Life Science, University of Trieste, Via L. Giorgieri, 10, 34127 Trieste, Italy; mrenzi@units.it; 4 Bioscience Research Center, Via Aurelia Vecchia, 32, 58015 Orbetello, Italy; tecla.bentivoglio@bsrc.it (T.B.); serena.anselmi@bsrc.it (S.A.); 5 School of Environment, Trent University, Peterborough, ON K9L 0G2, Canada

**Keywords:** atmosphere, biomonitoring, black locust, microfibres, tyre wear particles, Italy

## Abstract

**Simple Summary:**

This research investigated the presence of atmospheric microplastics, including those originating from tyre wear, in urban parks and rural roadside areas (Siena, Italy). We used the leaflets of the commonly found higher plant, *Robinia pseudoacacia* L. (black locust), as a novel biomonitor of atmospheric microplastic deposition. In the study, we observed that rural roadside locations had high concentration of tyre wear particles, whereas urban parks situated more than 500 m away from busy roads exhibited no detectable levels of tyre wear particles. In contrast, the number of plastic microfibres was significantly greater in urban parks, given their central city locations. The results suggest a substantial daily deposition of microplastics, potentially leading to human exposure in both residential and rural areas. In situations where human habitation is in proximity to roads, there is a heightened risk of exposure to tyre wear particles from road surfaces. Our results suggest that *Robinia pseudoacacia* L., with its widespread availability, waxy coating, and high surface-to-mass ratio, as well as ease of surface area determination for estimating deposition rates, can serve as a valuable resource for investigating the deposition of airborne microplastics including tyre wear particles.

**Abstract:**

Here we investigate the suitability of *Robinia pseudoacacia* L. (black locust) leaflets as a novel biomonitor of airborne microplastics (MPs) including tyre wear particles (TWPs). Leaflets were collected from rural roadside locations (ROs, *n* = 5) and urban parks (UPs, *n* = 5) in Siena, Italy. MPs were removed by washing, identified by stereomicroscope, and analysed for polymer type by Fourier transform infrared spectroscopy. Daily MP deposition was estimated from leaf area. The mass magnetic susceptibility and the bioaccumulation of traffic-related potentially toxic elements (PTEs) were also analysed. The total number of MPs at ROs was significantly higher at 2962, dominated by TWPs, compared with 193 in UPs, where TWPs were not found. In contrast, total microfibres were significantly higher in UPs compared with ROs (185 vs. 86). Daily MP deposition was estimated to range from 4.2 to 5.1 MPs/m^2^/d across UPs and 29.9–457.6 MPs/m^2^/d across ROs. The polymer types at ROs were dominated by rubber (80%) from TWPs, followed by 15% polyamide (PA) and 5% polysulfone (PES), while in UPs the proportion of PES (44%) was higher than PA (22%) and polyacrylonitrile (11%). The mean mass magnetic susceptibility, a proxy of the bioaccumulation of traffic-related metallic particles, was higher at ROs (0.62 ± 0.01 10^–8^ m^3^/kg) than at UPs (–0.50 ± 0.03 10^–8^ m^3^/kg). The content of PTEs was similar across sites, except for significantly higher concentrations of Sb, a tracer of vehicle brake wear, at ROs (0.308 ± 0.008 µg/g) compared with UPs (0.054 ± 0.006 µg/g). Our results suggest that the waxy leaflets and easy determination of surface area make *Robinia* an effective biomonitor for airborne MPs including TWPs.

## 1. Introduction

Microplastics (MPs, i.e., plastic particles < 5 mm) have become a significant global concern. They arise from photo- and biodegradation, mechanical breakdown (e.g., due to wind and friction), thermo-oxidative and hydrolytic degradation of plastic materials [1,2,3,4,5,6] and can be found in different shapes such as fibres, fragments, foams, pellets, and beads [7].

MPs have been detected in all environmental compartments, including aquatic [8,9,10,11,12] and terrestrial ecosystems [13,14,15,16], and the atmosphere [17,18,19,20,21,22]. Furthermore, MPs have been found in animal [23,24,25,26,27] and plant tissues [28,29], and even in human blood [30,31,32]. This widespread distribution raises serious concerns about the potential impacts of MPs on ecosystem and human health.

Synthetic textiles [33], the fragmentation of plastic products, and industrial emissions [34] are recognized as significant contributors to airborne MPs. Furthermore, tyre wear particles (TWPs), considering their chemical and physical characteristics, are classified as MPs [35]. The abrasion of tyre treads on road surfaces, with the consequent release of TWPs is an important source of airborne MPs [36,37].

It is known that plant leaves have a high capacity to intercept airborne particles from the atmosphere on their surface and can provide insights into the deposition levels of pollutants [38]. However, only a few studies have evaluated the deposition of MPs using leaves of higher plants such as shrubs [39], pine needles [40], tree species [41], and lettuce [42]. In addition, a number of studies have focused on the use of cryptogams such as mosses and lichens for monitoring the deposition of airborne MPs [43,44,45,46,47,48,49]. While some researchers have employed *Robinia pseudoacacia* L. leaves for investigating the deposition of potentially toxic elements (PTEs) from the atmosphere [50,51], no study has yet explored its potential for the analysis of atmospheric MPs, particularly regarding TWPs.

*Robinia,* a fast-growing tree species, has spread extensively in temperate regions of Europe, North America, and Southern Africa, earning its classification as an invasive allochthonous species due to shading and its capacity to modify soil conditions [52,53]. In the Mediterranean region, it has invaded a broad range of disturbed habitats, such as abandoned fields, arid locations, roadsides, and other ecosystems where the natural vegetation has undergone severe disturbances [52]. Its wide distribution, ease of identification, and that its leaves consist of leaflets that have a hydrophobic, waxy surface [54], potentially make it an effective biomonitor of atmospheric microplastic deposition.

This research explored the effectiveness of *Robinia* leaflets as a novel biomonitor to assess the deposition of airborne MPs. Leaflets were collected from urban parks and rural roadside locations reflecting opposite gradients in population and vehicle traffic densities. Samples were analysed for the accumulation and polymer composition of airborne MPs, and PTE content and magnetic analysis of traffic-related elements for source identification. To the best of our knowledge, this is the first study using *Robinia pseudoacacia* for the examination of airborne MPs, including TWPs.

## 2. Materials and Methods

### 2.1. Study Area

**Figure 1:**
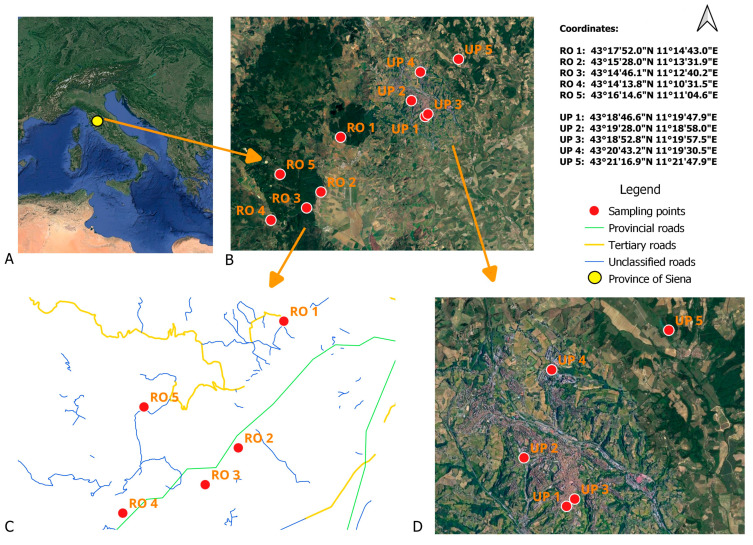
Study area (**A**); location of sampling sites: RO 1–5 = locations along rural asphalt roads, and UP 1–5 = urban parks (**B**); proximity of the RO sites to the nearest roads (**C**); and close-up of the city of Siena showing the UP sites (**D**).

The study was conducted in Siena and its outskirts (Tuscany, central Italy) at the end of the vegetation growing season (end of September 2022). The study sites included both urban parks in Siena (UPs, *n* = 5), situated away from major roads, and rural locations (ROs, *n* = 5) distant from human settlements and any other local sources of pollution, but conveniently located close to minor asphalt roads (Figure 1). The urban parks are visited by many locals and tourists during the spring–summer period, but vehicles are strictly prohibited in the city. In contrast, the trees at the roadside locations had an approximate distance of 5–10 m from the nearest provincial (RO 2, RO 3, RO 4), tertiary (RO 1), and unclassified roads (RO 5) that connect the small towns of the province of Siena. The city of Siena has a population of about 55,000 inhabitants, while the whole province has about 270,000 inhabitants. The elevation of the sampling sites ranged from 270 to 410 m asl. The climate is Mediterranean, with warm, dry summers; mean annual temperature is 13–14 °C and annual rainfall spans a range of 600–1000 mm.

### 2.2. Experimental Design

In this study, the common tree species *Robinia pseudoacacia* L. (black locust) was selected as a biomonitor for MP deposition, considering its widespread presence, and the high surface area and waxy coating of its leaves. At each study site, leaves of *R. pseudacacia*, each containing multiple leaflets, were collected from five trees closest to the centre of the site. After collection, all samples were stored in paper bags and frozen for subsequent analysis. In total, 50 samples were analysed for MPs by processing five replicates of 1 g of fresh leaflets from each site.

### 2.3. Microplastic Analysis

In the laboratory, MPs were extracted by washing each *Robinia* leaflet sample with 100 mL of deionized water inside beakers (ca. 10 fresh leaflets per 1 g sample). The leaflet samples were manually stirred in the deionised water for 3–4 min. The water samples were subsequently vacuum filtered onto cellulose filter papers (Watman Grade 1, 1001-090, 11 µm) with a diameter of 90 mm, and the filters stored in glass Petri dishes. The filter papers (*n* = 50, 1 filter paper per 100 mL) were examined for MPs under a stereomicroscope (Eurotek OXTL101TUSB equipped with an MDCE-5C digital camera) following a five-criteria method: unnatural colour, material homogeneity, particle resiliency, reflective surfaces, and limited fraying [43,44,45,46]. Fibres and fragments that met at least two criteria were considered anthropogenic and photographed [55], and further verified as plastic using a hot needle [56,57]. TWPs pose a challenge in terms of identification, as they do not exhibit a response to a hot needle. Therefore, they were categorized using specific criteria, including dark colour (black), elongated or cylindrical shape, rough surface texture, and rubbery flexibility when manipulated [47,58,59,60]. The length of all plastic particles was measured using the open-source image processing software ImageJ 1.53t.

### 2.4. Quality Control

To minimize the possibility of contamination, sampling and analysis were conducted with strict adherence to standard quality control procedures. Analytical process blanks were regularly processed to ensure the systematic management of laboratory contamination. Each piece of glassware underwent a triple rinsing with filtered deionized water prior to use and aluminium foil was used to cover all laboratory glassware during the extraction of MPs to prevent airborne contamination. Surfaces were cleaned using a combination of paper towels and filtered deionized water. Furthermore, individuals wore cotton clothing throughout the procedure. A washing process blank containing only the filtered deionized water that was used to wash the leaflet samples was carried out for each site. These blanks remained open throughout the leaflet washing procedure and did not contain any substantial presence of MPs falling within the range of 0–1 MPs per blank.

### 2.5. Estimation of MP Deposition Rates

The MP deposition period spanned approximately five months from full leaf emergence at the end of April to the end of the growing season at the end of September. The leaflets were photographed, and their upper surface area was calculated using the image software Leafscan 2.1.1 [61]. The average daily MP deposition rate was estimated based on the accumulation of MPs on the leaflets and an exposure period of 150 days (5 months) [45] according to the formula:

MP deposition (MPs m^–2^ d^–1^) = MP concentration (MPs g dw^–1^) × sample mass (g dw^–1^ m^–2^)/150 (d).

There was no occurrence of heavy rainfall during the study period that could potentially result in the particles being washed away [62].

### 2.6. Characterization of Microplastics

Leaf samples were treated in a glove box fitted with a HEPA filter to prevent contamination. Further, positive and negative controls were performed (*n* = 3) to ensure the quality of the entire analytical process. Extraction was performed by digesting the samples with 30% H_2_O_2_ solution and the filtered, digested samples were analysed using a stereomicroscope at 10–80× (SMZ-800 N; NIS-elements D software 5.11.03, Nikon, Tokyo, Japan). The polymer composition of the target particles was investigated by microscopy combined with micro-Fourier transform infrared spectroscopy (Nicolet iN10 MX, ThermoFischer Scientific, Waltham, MA, USA) using an MCT-A detector—cooled with liquid nitrogen and operated in reflection mode (spectral range between 7800 and 650 cm^–1^). Identification was performed by determining the spectral match of target elements (%) with microplastic spectral libraries (OMNIC™ Picta™ Software 1.7.192 Libraries, ThermoFisher Scientific, Waltham, MA, USA) integrated with internal spectral libraries containing TWP spectra. A threshold for spectra acceptance was set at a match of >80%. The detection threshold for particle size was 10 μm.

### 2.7. Metal Content and Magnetic Analysis

The leaflets were further analysed for traffic-related PTE content and magnetic properties to support source identification. Plant leaflets were oven-dried at 40 °C for 24 h, digested in 3 mL of 70% HNO_3_ and 0.5 mL of 30% H_2_O_2_ using a microwave-digestion system (Milestone Ethos 900, Bergamo, Italy) at 280 °C and 55 bar. The content of traffic-related PTEs (namely, Fe, Al, Cu, Zn, Ba, Cr and Sb) was then quantified by ICP-MS (Sciex Elan 6100, Perkin Elmer, Waltham, MA, USA). Analytical quality was verified using the certified reference material GBW 07603 “plant leaves”; recoveries were in the range 83–127%; the precision of the analysis was expressed by the relative standard deviation of three replicates and was within 10% for all elements. The results are expressed on a dry weight basis.

For the magnetic analysis, dry samples were placed into standard 8 cm^3^ plastic cubes, and mass magnetic susceptibility (χ, m^3^ kg^–1^) was determined with an Agico KLY5 m after measuring the net weight of the sample.

### 2.8. Statistical Analysis

The differences in the concentration of MPs, PTEs, and magnetic susceptibility between the RO and UP sites were assessed using the Mann–Whitney U test (*p* < 0.05). Statistical analysis was carried out using the open-source software R 4.3.1 [63].

## 3. Results

**Figure 2:**
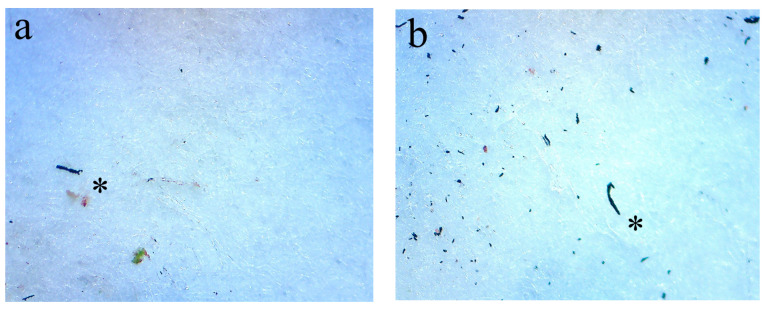

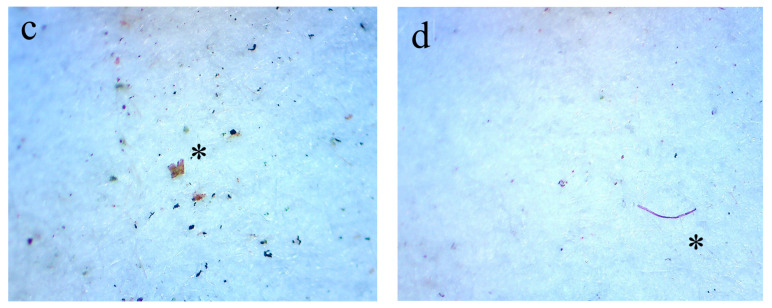
Images of tyre wear particles (TWPs) in samples from rural roadside locations and fragments and fibres from urban parks under a stereomicroscope (tyre wear particle L = 252 µm (**a**); tyre wear particle L = 398 µm (**b**); black fragment L = 239 µm (**c**); purple fibre L = 661 µm (**d**); L—length, see the asterisk).

In total, 3155 MPs were found across all sites, 193 in UPs and 2962 at ROs (*p* < 0.05; Table 1), the latter mainly due to the remarkable abundance of TWPs (Figure 2). Within the RO sites, 2872 TWPs were found, whereas no instance of TWPs was detected in UPs. The quantity of plastic fibres and fragments was notably lower at ROs compared to UP; 86 vs. 185 (*p* < 0.05) and 4 vs. 8, respectively (Figure 2). The daily deposition of MPs at the RO sites was significantly higher than at the UP sites (Table 1), consistent with the high amount of TWPs.

**Figure 3:**
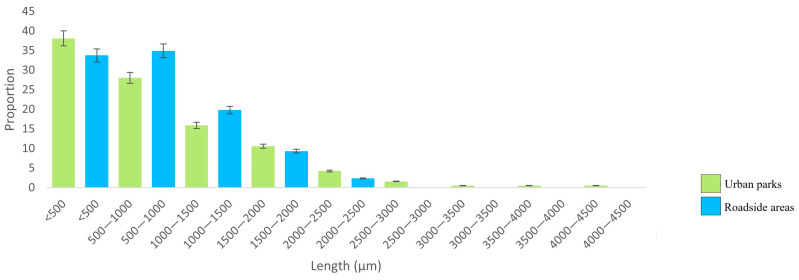
Distribution of microfibre lengths (µm; *n* = 271) in urban parks and rural roadside locations.

**Figure 4:**
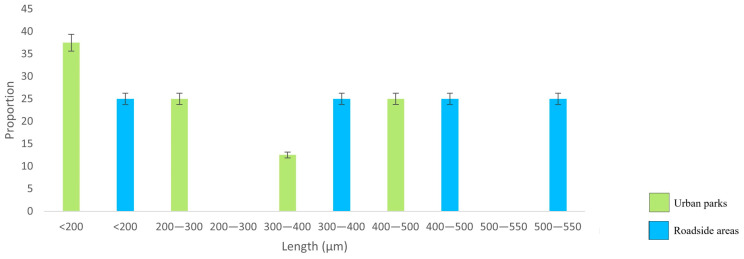
Distribution of fragment lengths (µm; *n* = 12) in urban parks and rural roadside locations.

**Figure 5:**
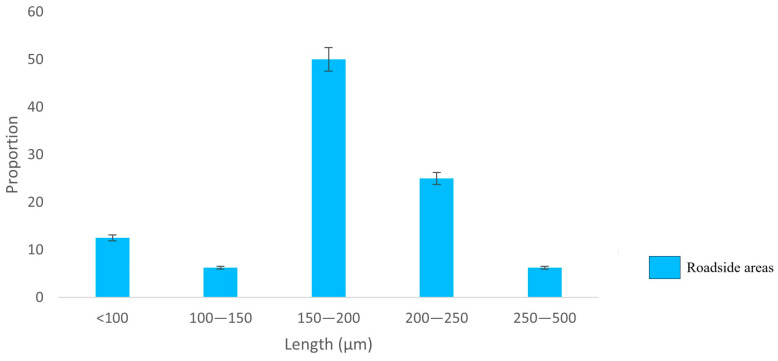
Distribution of tyre wear particle lengths (µm; *n* = 2872) in rural roadside locations.

The proportion of total fibres showed a clear decrease with increasing fibre length, with shorter fibres being predominant in both the UP and RO locations; longer fibres were found only at the UP sites (Figure 3). At the ROs, the fibre length ranged from 173 to 2272 µm, while in UPs, the range was 129–4335 µm. In contrast, fragment length (Figure 4) did not show any pattern at either the RO or UP sites. The TWP size class of 150–200 µm was predominant, with a frequency distribution approaching a Gaussian curve (Figure 5), although the distribution may reflect the limits of particle identification at <150 µm.

**Figure 6:**
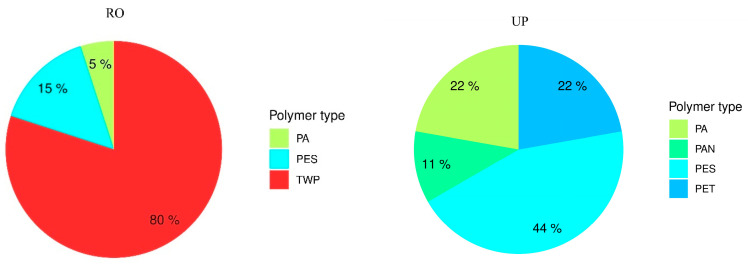
Proportions of polymer types (TWP—tyre wear particles; PA—polyamide; PAN—polyacrylonitrile; PES—polysulofone; PET—polyethylene terephthalate) at the RO (rural roadside) and UP (urban parks) locations.

Qualitatively, at the RO locations TWP proportions (Figure S1) were higher than polyamide (PA) and polysulfone (PES) at 80%, 15%, and 5%, respectively (Figure 6). In contrast, at the UP sites the proportion of PES was higher than PA, polyethylene terephthalate (PET) and Polyacrylonitrile (PAN), with proportions of 44%, 22%, 22%, and 11%, respectively (Figure 6).

There was no significant difference in the concentration of Fe, Al, Cu, Zn, and Cr between the RO and UP sites, while Sb concentrations were significantly different, with higher values at the ROs than in UPs; mean concentration of 0.308 ± 0.008 mg kg^–1^ and 0.054 ± 0.006 mg kg^–1^, respectively (Table 2). Mass magnetic susceptibility was higher at the RO locations compared with the UP sites, with mean values of −0.50 ± 0.03 10^−8^ m^3^ kg^–1^ in UPs and 0.62 ± 0.01 10^−8^ m kg^–1^ in ROs (Table 2).

## 4. Discussion

This study showed that the RO sites experienced a notable deposition of TWPs owing to the wearing of vehicle tyres with consequent settling on nearby vegetation. At the RO sites, *Robinia* leaves were collected at 5–10 m from the roads due to the fact that this species is not native and does not penetrate deep into the local vegetation, so even at remote sites, *Robinia* trees are only available close to roads. Conversely, TWPs were not found at the UP sites, due to their central city location where traffic is very limited, thus being at a considerable distance (>500 m) from busy roads. Consequently, ref. [64] it can be reported that the amount of TWPs collected in atmospheric dry deposition decreased as the distance from roads increased.

In contrast, the number of plastic fibres was significantly higher at the UP sites. A likely explanation is that the UP sites are surrounded by residential areas and are frequently visited by people, especially during the summer months, while the RO sites are distant from residential areas, irrespective of their proximity to roads. Likewise, there was a greater abundance of fragments in UP sites, mirroring the pattern seen with the number of fibres, suggesting the impact of human habitation was a potential causal factor. In line with the overall MP accumulation at both sites, the daily MP deposition rate was significantly higher at the RO sites, underscoring that if MPs are present in remote locations, in cases of human habitation close to roads, there would be substantial daily exposure due to the presence of TWPs. However, when excluding traffic-related MPs, the daily deposition of MPs was greater at the UP sites, primarily owing to the higher abundance of fibres. This is consistent with a study that identified a greater daily deposition of MPs in urban parks located in Milan, Italy, compared to a remote site situated 50 km away using lichen transplants during a 3-month exposure (43–50 vs. 21–43 MPs m^–2^ d^–1^; [45]). In another study, the daily deposition of MPs ranged from 21 to 60 MPs m^–2^ d^–1^ in three different urban locations of Southern Ontario, Canada, using moss bags as biomonitors during a 45-day exposure [47]. These studies reported a higher deposition during a shorter timeframe than our study. This suggests that plant leaves may be less efficient in capturing MPs on their surfaces, possibly serving as a temporary sink compared to lichens and mosses. Likewise, ref. [39] it has been suggested that higher plants act as temporary storage for microplastics, given the dynamic pattern of accumulation and loss of MPs on plant surfaces.

Considering that Sb is a well-known indicator of non-exhaust (brake wear) traffic emissions, the higher concentrations of Sb observed at the RO sites, consistent with TWPs, can be attributed to vehicle traffic. It is noteworthy that [65] Sb is found in the chemical content of the tyre rubber. However, the chemical composition of rubber particles may be influenced by factors such as tyre type and vehicle specifications [36].

Despite their relatively low values, the mass magnetic susceptibility switched from negative values (diamagnetism) at the UP sites to positive magnetic susceptibility values for all the RO sites (up to 2.80 ± 0.013 m^3^ kg^–1^), excluding one highlighting the modest bioaccumulation of ferromagnetic particles that are usually linked to non-exhaust metallic emissions in traffic-related contexts, as found by [66,67].

While there was no statistically significant difference in fibre and fragment length between the UP and RO sites, it is worth noting that the longest fibres were detected at the UP sites. This suggests that fibre length was influenced by proximity to source, as the UP sites were surrounded by inhabited areas. Nonetheless, the fact that both sites primarily featured short fibres can be attributed to size fractionation during atmospheric transport of MPs, which tends to result in a reduction in fibre size with distance travelled [45,46]. Although the dimensions of TWPs are determined by many factors, including tyre material, age, type of road, driving conditions, vehicle weight, etc. [68], the almost Gaussian frequency distribution of TWP length at the RO sites is a further confirmation that the impact of vehicle traffic was only local.

The sites at UPs had a greater diversity of polymer types, in contrast to the RO sites, which were dominated by TWPs with an average of 574.4 ± 310.4 particles per sample. The lowest number of particles was found in the analytical sample RO 1 with 48 particles, while sample RO 4 had the highest proportion of TWPs with 792 particles, which likely reflects a number of factors such as traffic density, road type, and position (e.g., road curvature) and the distance of individual trees from the road.

## 5. Conclusions

Despite their rural nature, the RO locations exhibited a remarkably (significantly) higher abundance of MPs compared with the UP sites located in the city centre of Siena. This disparity was due to the substantial presence of TWPs at the RO sites caused by the impact of local vehicle traffic, as suggested by chemical and magnetic analysis. In contrast, the UP sites showed higher counts of fibres, especially longer fibres, owing to their proximity to inhabited areas.

Overall, this study suggests that *Robinia*, with its widespread availability, waxy coating, and high surface-to-mass ratio, as well as ease of surface area determination for deposition rates, may serve as a valuable resource for investigating the deposition of airborne MPs, especially TWPs.

## Figures and Tables

**Table 1:** Microplastics count (mean ± standard deviation) for total, fibre, fragment, and tyre wear particles (TWP) found on *Robinia* leaflets and daily MP deposition (MPs m^–2^ d^–1^) at ROs (rural roadside) and UPs (urban parks) locations.

Site	Total MPs (*n*)	Fibre (*n*)	Fragment (*n*)	TWP (*n*)	Mean Fibre Length (µm)	Mean TWP Length (µm)	Daily MP Deposition (MPs m^–2^ d^–1^)
**RO 1**	51.8 ± 1.64	3.80 ± 1.64	0.00 ± 0.00	48 ± 0.15	612 ± 370	50.03 ± 7.50	29.86 ± 0.95
**RO 2**	543.2 ± 2.17	4.80 ± 1.29	0.40 ± 0.55	538 ± 80.70	1073 ± 500	115.37 ± 36.14	313.14 ± 1.25
**RO 3**	741.4 ± 2.41	3.00 ± 2.00	0.40 ± 0.55	738 ± 110.70	1037 ± 501	176.40 ± 15.00	427.39 ± 1.39
**RO 4**	793.8 ± 0.45	1.80 ± 045	0.00 ± 0.00	792 ± 118.80	512 ± 337	222.75 ± 19.85	457.60 ± 0.26
**RO 5**	759.8 ± 2.59	3.80 ± 2.59	0.00 ± 0.00	756 ± 113.40	647 ± 414	368.05 ± 169.63	438.00 ± 1.49
**UP 1**	7.20 ± 3.49	7.00 ± 3.54	0.20 ± 0.45	–	881 ± 767	–	4.15 ± 2.01
**UP 2**	7.20 ± 3.03	6.80 ± 2.68	0.40 ± 0.89	–	1080 ± 922	–	4.15 ± 1.75
**UP 3**	8.00 ± 2.12	7.80 ± 1.79	0.20 ± 0.45	–	930 ± 555	–	4.61 ± 1.22
**UP 4**	8.80 ± 4.71	8.60 ± 4.70	0.20 ± 0.45	–	892 ± 468	–	5.07 ± 2.72
**UP 5**	7.40 ± 2.88	6.80 ± 3.42	0.60 ± 0.55	–	994 ± 743	–	4.27 ± 1.66

**Table 2:** Concentration (mean ± standard deviation, mg kg^–1^ dw) of potentially toxic elements (Fe, Al, Cu, Zn, Ba, Cr, and Sb) and magnetic susceptibility (χ, mean ± standard deviation, 10^−8^ m^3^ kg^–1^) in leaflets of *Robinia pseudoacacia* L.

Site	Fe	Al	Cu	Zn	Cr	Sb	Ba	χ, kg
**RO 1**	103 ± 1	102 ± 1	8.2 ± 0.1	25.8 ± 0.6	0.6 ± 0.3	0.71 ± 0.02	17.8 ± 0.2	−0.548 ± 0.0136
**RO 2**	168 ± 4	117 ± 2	7.5 ± 0.3	28.2 ± 0.9	0.8 ± 0.1	0.20 ± 0.01	17.8 ± 0.1	0.670 ± 0.009
**RO 3**	152 ± 4	88 ± 0.4	7.6 ± 0.1	22.2 ± 1.0	0.5 ± 0.1	0.24 ± 0.002	20 ± 0.1	0.021 ± 0.0099
**RO 4**	181 ± 2	146 ± 3	8.1 ± 0.1	22.9 ± 0.1	0.5 ± 0.1	0.30 ± 0.01	17.7 ± 0.1	2.80 ± 0.01
**RO 5**	209 ± 2	162 ± 2	6.9 ± 0.1	18.8 ± 0.6	0.7 ± 0.1	0.10 ± 0.01	16.2 ± 0.1	0.155 ± 0.0096
**UP 1**	170 ± 3	181 ± 6	6.3 ± 0.1	14.2 ± 0.4	0.6 ± 0.1	0.10 ± 0.01	12.9 ± 0.2	−0.233 ± 0.0202
**UP 2**	97 ± 1	136 ± 1	9.2 ± 0.1	24.8 ± 0.5	0.3 ± 0.1	0.04 ± 0.01	7.4 ± 0.04	−0.643 ± 0.0156
**UP 3**	134 ± 2	92 ± 1	6.0 ± 0.2	20.6 ± 0.6	0.6 ± 0.1	0.10 ± 0.01	11.3 ± 0.1	−0.543 ± 0.0298
**UP 4**	87 ± 2	87 ± 1	8.0 ± 0.1	22.1 ± 0.5	0.3 ± 0.1	0.10 ± 0.01	6.4 ± 0.1	−0.555 ± 0.046
**UP 5**	160 ± 8	76 ± 1	7.7 ± 0.1	16.0 ± 0.5	0.4 ± 0.1	0.10 ± 0.01	33.6 ± 0.1	−0.508 ± 0.0341

## Data Availability

Data are contained within the article.

## Supplementary Materials

The following supporting information can be downloaded at: https://www.mdpi.com/article/10.3390/biology12121456/s1, Figure S1: FTIR spectra example of tyre wear particles found during the polymer characterization using a spectral library with a match of 78.39%.
