# Supplementary material for: Robinia pseudoacacia L. (Black Locust) Leaflets as Biomonitors of Airborne Microplastics
Source: Biology (Basel). 2023 Nov 22;12(12):1456. doi: 10.3390/biology12121456 (PMC10740701; doi:10.3390/biology12121456)
Supplement: Supplementary file 1 [file biology-12-01456-s001.zip › biology-2709815-supplementary.pdf]

biology

MDPI

Supplementary Material

Robinia pseudoacacia L. (black locust) leaflets as biomonitors of airborne microplastics

Mehriban Jafarova[1*], Lisa Grifoni[1,2], Monia Renzi[3], Tecla Bentivoglio[4], Serena Anselmi[4], Aldo Winkler[2], Luigi Antonello Di Lella[1], Lilla Spagnuolo[2], Julian Aherne[5], Stefano Loppi[1]

[1] Department of Life Sciences, University of Siena, Siena 53100, Italy; mehriban.jafarova@student.unisi.it (M.J.); l.grifoni2@student.unisi.it (L.G.); luigi.dilella@unisi.it (L.A.L.); stefano.loppi@unisi.it (S.L.)
[2] Istituto Nazionale di Geofisica e Vulcanologia, 00143 Rome, Italy; aldo.winkler@ingv.it (A.W.); lilla.spagnuolo@ingv.it (L.S.)
[3] Department of Life Science, University of Trieste, via L. Giorgieri, 10, Italy; mrenzi@units.it (M.R.)
[4] Bioscience Research Center, via Aurelia Vecchia, 32, Italy; tecla.bentivoglio@bsrc.it (T.B.); serena.anselmi@bsrc.it (S.A.)
[5] School of Environment, Trent University, Peterborough, ON K9L 0G2 Canada; jaherne@trentu.ca (J.A.)
* Correspondence: mehriban.jafarova@student.unisi.it (M.J.)

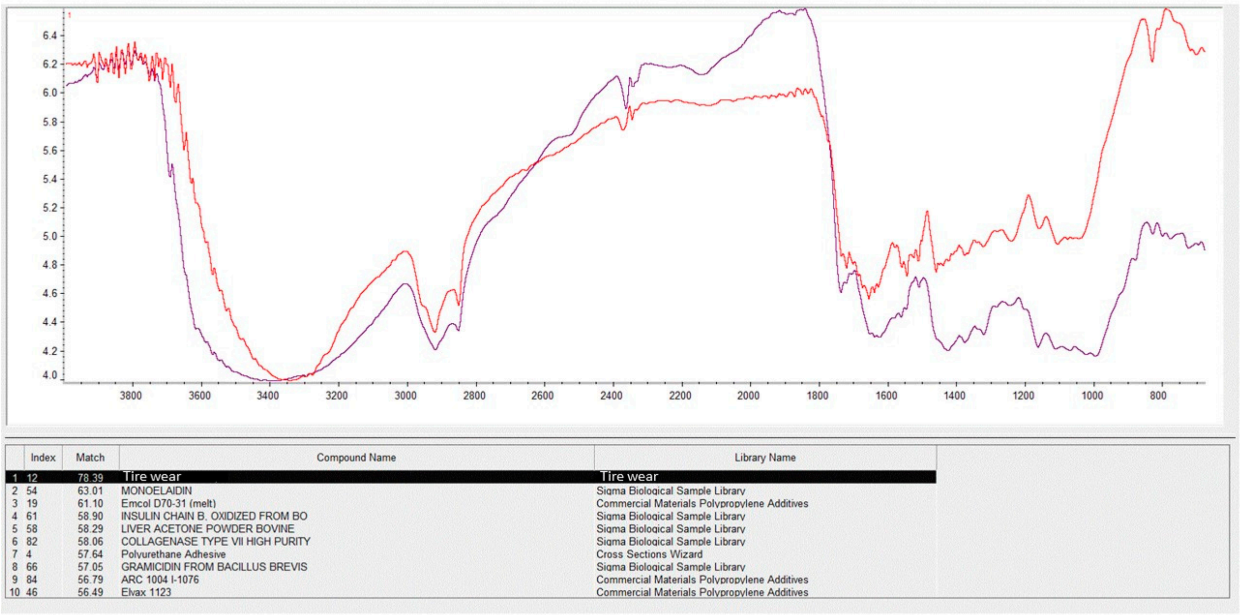

Figure S1. FTIR spectra example of tire wear particles found during the polymer characterization using a spectral library with a match of 78.39 %.
